# Cardiovascular risk profile in individuals initiating treatment for overactive bladder – Challenges and learnings for comparative analysis using linked claims and electronic medical record databases

**DOI:** 10.1371/journal.pone.0205640

**Published:** 2018-10-16

**Authors:** E. Vonesh, K. L. Gooch, V. Khangulov, C. R. Schermer, K. M. Johnston, S. M. Szabo, J. S. Rumsfeld

**Affiliations:** 1 Department of Biostatistics, Northwestern Medicine, Chicago, IL, United States of America; 2 Medical Affairs, Astellas Pharma USA, Northbrook, IL, United States of America; 3 Boston Strategic Partners, Boston, MA, United States of America; 4 Broadstreet HEOR, Vancouver, BC, Canada; 5 Faculty of Medicine, University of Colorado, Denver, CO, United States of America; Biomedical Research Foundation, Academy of Athens, GREECE

## Abstract

For managing overactive bladder (OAB), mirabegron, a β3 adrenergic receptor agonist, is typically used as second-line pharmacotherapy after antimuscarinics. Therefore, patients initiating treatment with mirabegron and antimuscarinics may differ, potentially impacting associated clinical outcomes. When using observational data to evaluate real-world safety and effectiveness of OAB treatments, residual bias due to unmeasured confounding and/or confounding by indication are important considerations. Falsification analysis, in which clinically irrelevant endpoints are tested as a reference, can be used to assess residual bias. The objective in this study was to compare baseline cardiovascular risk among OAB patients by treatment, and assess the presence of residual bias via falsification analysis of OAB patients treated with mirabegron or antimuscarinics, to determine whether clinically relevant comparisons across groups would be feasible. Linked electronic health record and claims data (Optum/Humedica) for OAB patients in the United States from 2011–2015 were available, with index defined as first date of OAB treatment during this period. Unadjusted characteristics were compared across groups at index and propensity-matching conducted. Falsification endpoints (hepatitis C, shingles, community-acquired pneumonia) were compared between groups using odds ratios (ORs) and 95% confidence intervals (CI). The study identified 10,311 antimuscarinic- and 408 mirabegron-treated patients. Mirabegron patients were predominantly older males, with more comorbidities. The analytic sample included 1,188 antimuscarinic patients propensity-matched to 396 mirabegron patients; after matching, no significant baseline differences remained. Estimates of falsification ORs were 0.7 (CI:0.3–1.7) for shingles, 1.5 (CI:0.3–8.2) for hepatitis C, 0.8 (CI:0.4–1.8) and 0.9 (CI:0.6–1.4) for pneumonia. While propensity matching successfully balanced observed covariates, wide CIs prevented definitive conclusions regarding residual bias. Accordingly, further observational comparisons by treatment group were not pursued. In real-world analysis, bias-detection methods could not confirm that differences in cardiovascular risk in patients receiving mirabegron versus antimuscarinics were fully adjusted for, precluding clinically relevant comparisons across treatment groups.

## Introduction

Overactive bladder (OAB) is characterized by urge urinary incontinence and urgency, nocturia, and high urinary frequency. The prevalence of OAB has been estimated to be 11.8% in the United States (US), with higher rates in older individuals.[1] While behavioral modifications including bladder training, pelvic floor training, and limitation of fluids are intended as the first line of treatment for OAB, pharmacologic intervention is a mainstay of OAB management.[2] To date, antimuscarinic therapies–including oxybutynin, tolterodine, solifenacin, flavoxate, fesoterodine, trospium, or darifenacin–have been the most common first-line pharmacologic treatment for OAB.[3] Mirabegron (Myrbetriq/Betmiga; Astellas Pharma) is a β3 adrenergic receptor agonist with demonstrated efficacy and safety in managing the symptoms of OAB.[4] In clinical practice, mirabegron is typically given as second-line pharmacotherapy after discontinuation or failure of therapy with antimuscarinics.[3] While clinical trials have shown mirabegron to be both efficacious and safe,[4] in two randomized, placebo-controlled studies of healthy volunteers, mirabegron was associated with dose-related increases in supine blood pressure with the currently marketed and maximum recommended dose of 50 mg.[5] The mean increase in systolic blood pressure (SBP)/diastolic blood pressure (DBP) was approximately 3.5/1.5 mm Hg greater than placebo. In three, phase 3, 12-week, double-blind, placebo-controlled, safety and efficacy studies of OAB patients receiving mirabegron 25 mg, 50 mg or 100 mg once daily, mean increases in SBP/DBP of approximately 0.5–1.0 mm Hg were observed compared to placebo.[5] Both SBP and DBP increases were reversible upon discontinuation of treatment.[5]

It is important to determine whether findings from randomized controlled studies are also observed in a real-world setting. In a real-world setting, integrated electronic health record (EHR) and claims data can also provide confirmation of dispensed–as opposed to prescribed–medications, as well as details of baseline cardiovascular risk profiles (e.g. vital signs, smoking status) that are not typically captured in billing claims but are required to inform any necessary statistical adjustments. To date, a real-world assessment of cardiovascular risk in OAB patients has not been conducted.

A key challenge in this setting is the potential for residual bias in observational data due to unmeasured confounders and/or confounding by indication, e.g. if patients receiving any OAB treatment are systematically different from patients receiving alternative therapies, or those who are untreated. Addressing residual bias is critical when using observational data to probe comparative outcomes.[6] In previous database analyses it was found that OAB patients initiating mirabegron tended to be older at treatment initiation, with a greater comorbidity burden and higher healthcare resource utilization, compared to those initiating treatment with antimuscarinics.[3] This is likely a result of mirabegron’s typical positioning as a second-line pharmacological agent after failure of antimuscarinics.[3]

The overarching aim of the study was, using the most appropriate statistical methodology for mitigating residual bias, to compare baseline cardiovascular risk profiles of OAB patients initiating antimuscarinics vs. mirabegron, and determine comparative cardiovascular outcomes, such as blood pressure change, between treatment groups. However, prior to undertaking that investigation, the team planned an unbiased a priori assessment of the feasibility of that comparative study, with a particular focus on determining whether potential residual bias could be present due to unmeasured confounding, including differences in prior treatment patterns and treatment switching. Should the initial feasibility assessment satisfy the study team that a rigorous study could be conducted, a larger outcome comparative analysis would then be undertaken. The objective of the study described here is to compare cardiovascular risk profiles of OAB patients initiating antimuscarinics vs. mirabegron; and to present the findings of the feasibility assessment for the comparative study of cardiovascular outcomes across treatment groups that the cardiovascular risk profiles informed.

## Methods

### Study design and patient population

The study was designed as a real-world, US-based retrospective cohort study of patients receiving treatment for OAB. Data were available from October 2011 to June 2015.

The overall study population was derived from all individuals diagnosed with OAB, based on the International Classification of Diseases-9^th^ Edition (ICD-9) codes that indicate a diagnosis of OAB (ICD-9 596.65,596.51,788.3,788.31,788.33,788.41,788.43,788.63,788.91). While there is no specific ICD-9 code for OAB, the proposed ICD-9 codes above are consistent with previously published research that evaluated OAB patients using real-world datasets.[7–10]

For the feasibility assessment, patients were eligible for inclusion based on dispensation billing records for mirabegron or an antimuscarinic therapy (oxybutynin, solifenacin, tolterodine, flavoxate, fesoterodine, trospium, darifenacin) between October 2012 and December 2014 (“identification period”). Both EHR records of prescriptions written and billing claims for dispensed prescriptions were initially considered for patient identification, however due to a large discrepancy indicating a high frequency of unfilled prescriptions (more than twice as many prescriptions were identified in the EHR versus the claims data), claims billing records were ultimately used to determine eligibility. Index date was defined as first prescription dispensation during the identification period. Patients with a diagnosis for OAB without a billing record for mirabegron or an antimuscarinic were included in the untreated cohort as of their first OAB-related health claim during the identification period. Data from October 2011 onwards were used to characterize medical and treatment history for each patient in the 12 months prior to index date. Follow-up included time from index date through June 2015 with a minimum potential follow-up of six months for those patients having an index date in December 2014. Follow-up times were censored for those individuals who died or left the claims database due to changing coverage. Patients were included in the mirabegron or antimuscarinic treatment group based on the first treatment they received during the identification period, noting that treatment switching after index date may have occurred, and that patients may have received other OAB therapies prior to the identification period.

Further eligibility criteria required that patients:

- Had at least 12 months of continuous coverage in the claims data prior to index date in order to comprehensively assess risk factors and comorbidities at baseline;
- Were 18 years of age or older and have at least one baseline blood pressure (measured and reported using methodologies per usual clinical practice) recorded in the EHR within 90 days prior to index date.

The criteria for exclusion were:

- Pregnancy during the study period;
- Received combination therapy of mirabegron and an antimuscarinic at index date;
- Received pharmacologic therapy for OAB during the year prior to the identification period but remained untreated for OAB during the identification period;
- Had a recorded cardiovascular event (myocardial infarction, unstable angina, cardiovascular death, cerebrovascular accident, peripheral arteriopathy, aortic event, heart failure, coronary artery bypass grafting, atrial fibrillation, transient ischemic attack, percutaneous intervention, angioplasty)[11] within 30 days prior to the index date.

If the feasibility assessment indicated it reasonable to proceed with the cardiovascular outcomes study, the goal of those analyses would be to determine whether differences in SBP and/or DBP changes occur in patients receiving mirabegron compared to antimuscarinics, and the association of those changes with cardiovascular events. It was therefore also important to perform sample size calculations *a priori*, to determine whether the available number of patients meeting the inclusion and exclusion criteria would be sufficient, if the outcome of the feasibility assessment to identify residual bias justified proceeding with the cardiovascular outcomes study. Details of the calculations are included in Appendix A. At power of 0.80, a sample of 500 mirabegron patients and 1500 antimuscarinic patients would be required to detect a systolic blood pressure difference of 2.5 mmHg assuming a 14 mmHg standard deviation. When based on a power of 0.90, the sample size increased to 645 mirabegron patients and 1935 antimuscarinic patients.

All analyses were conducted in SAS version 9.4.

### Data source

The study utilized an Optum integrated claims billing and EHR dataset from the US. The Optum claims dataset is widely used for pharmacoepidemiologic, pharmacoeconomic, and outcomes research studies in a variety of diseases,[12, 13] including cardiovascular diseases [14] and OAB.[15] The Optum dataset includes the eligibility, medical, and pharmacy claims data from United Health, a large commercial health plan affiliated with Optum. The individuals included within this health plan are geographically diverse, from across the US, comprising approximately 3 to 4% of the US population. The database includes data from 2003 to 2015 and has almost 13 million registrants annually.

Optum claims data were integrated with Humedica primary care EHR data for the subset of the Optum OAB population included in both datasets. Inclusion in the Humedica EHR is based on physician participation in the network. Reported EHR data include medications, laboratory results, vital signs, demographics, hospitalizations, outpatient visits, and physician notes. Claims data linked to the EHR can be used to identify those prescriptions that were actually dispensed, indicating that identified patients filled a prescription for the study medication. Hence medication use in this study was defined by claims rather than EHR data. By linking the Humedica EHR data to Optum claims data, it is possible to identify prescriptions filled by patients, along with cost and charge amounts associated with all covered healthcare utilization.

### Bias reduction

Initial unadjusted comparisons conducted for the feasibility assessment were descriptive in nature and statistical comparisons were not made across treatment groups. A propensity score analysis was undertaken to mitigate the effects of bias within the observational data source. A logistic regression model was fit to characterize the likelihood (i.e., propensity score) of an individual being in the mirabegron treatment group, while adjusting for a range of demographic (age, sex, ethnicity, health plan type, geographic region variables) and clinical (smoking status, BMI, blood pressure, cholesterol, cardiovascular history, comorbidities, concomitant medications) variables. It was anticipated *a priori* that there would be substantially more antimuscarinic patients than mirabegron patients available in the dataset and that an n:1 matching algorithm would make most efficient use of the available data. Based on exploratory review of the eligible population sizes, antimuscarinic patients were propensity score matched to mirabegron patients in a 3:1 manner using a greedy matching algorithm to form the analytic sample.[16]

### Quality assessment and falsification analysis

An assessment of data quality and completeness was required prior to undertaking further analyses. Due to the nature of US health insurance, OAB patients can enter and leave the enrollment plan over time, whereby a hiatus in coverage could be observed in either one or both data sources. Quality assessment of the data focused on a test sample of individuals untreated for OAB and included: comparison of all blood pressure, cholesterol, age, and BMI against plausible ranges (overall and stratified by age <65 vs. ≥ 65 and sex); and rates of missing values in variables included in the propensity score model. Within the treated cohorts, additional quality checks included tabulation of censoring from the cohort and reasons for drop out; assessment of overlap and gaps across EHR and claims data; and assessment of treatment switches across antimuscarinics and mirabegron following index date, which enabled characterization of the proportion of follow-up time that a mirabegron patient was exposed to antimuscarinics and vice-versa.

While numerous analytic methods are available to address confounding by indication, adequately minimizing bias may not be feasible, particularly in the case of unmeasured confounders.[17–19] Falsification analysis is a method that has recently been proposed for assessing the potential for residual bias in analyzing a specific research question in an observational data source.[20, 21] Within a falsification analysis, an endpoint thought to be unrelated to the exposure of interest is pre-specified, and the association between this outcome and the exposure is tested after statistical adjustments have been made. Any spurious residual association observed between the exposure and the falsification outcome suggests that bias may be present within the data, and additional analyses are not recommended unless this bias can be explicitly addressed.

Falsification endpoints with no known association with either medication class under study were pre-specified by clinical experts, and included shingles (ICD-9 053[22]), hepatitis C virus (ICD-9 070.44[23]), and community-acquired pneumonia (ICD-9 480.x-486.x[24]). For each falsification endpoint, odds ratios and corresponding 95% confidence intervals (CIs) of the association between treatment and outcome were calculated for the mirabegron and antimuscarinic propensity-matched cohorts.

## Results

**Fig 1:**
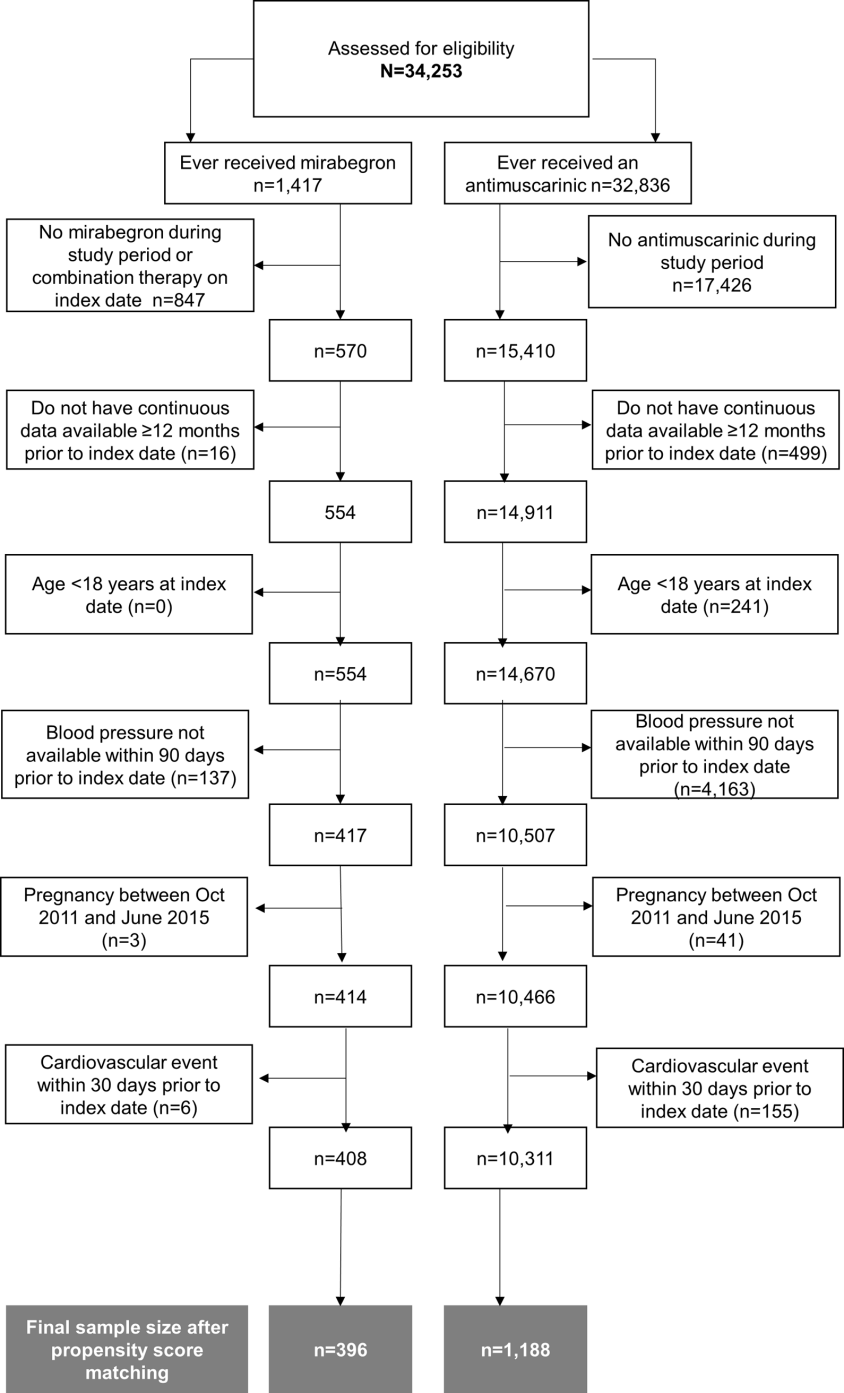
Cohort attrition.

To derive the study sample, 34,243 individuals (1,417 ever treated with mirabegron and 32,836 ever treated with an antimuscarinic) were initially identified for potential inclusion (Fig 1), based on a diagnosis of OAB and a billing record for a dispensed prescription at any point in time (e.g. not necessarily during the study period). It is of interest to note that more than twice as many patients had a record of a written prescription for mirabegron or an antimuscarinic in the EHR (data not shown) without a record of a prescription being dispensed in the claims data, perhaps indicating concerns with primary adherence and potential for bias in inducing differences across treatment groups. More than half of antimuscarinic patients (n = 17,426) and approximately one third of mirabegron patients (n = 847) were excluded because they were not dispensed a prescription during the study follow-up period (i.e. while these individuals received the medication of interest at some point during data coverage, they did not have a filled prescription during the study period). Of the resulting 15,980 patients (15,410 who received an antimuscarinic during the study period and 570 receiving mirabegron), most had 12 months’ continuous data available prior to the index date in at least one of the EHR or claims databases, and were at least 18 years of age, with only a small number of exclusions related to these criteria (n = 16 exclusions for mirabegron and n = 740 exclusions for antimuscarinics). The requirement of a blood pressure measure being available within 90 days of index date resulted in 137 exclusions in the mirabegron arm and 4,163 exclusions in the antimuscarinic arm. A small number of exclusions were made due to recent pregnancy and/or cardiovascular events. After applying all inclusion and exclusion criteria, the final sample was reduced to 408 mirabegron patients and 10,311 antimuscarinic patients. The antimuscarinic group was then further reduced to create a 3:1 propensity-matched sample to mirabegron. After 3:1 propensity matching, the final sample size was 396 in the mirabegron group and 1,188 in the antimuscarinic group. Thus, of the 15,980 OAB patients who received a prescription during the study period, approximately ten percent were eligible for the final analytic study population.

During the quality assessment stage, baseline blood pressure measurements were available for most individuals in the untreated group (77.7%), with less than 0.5% of these data flagged as implausible or likely data entry errors. Women were more likely to have recorded blood pressure measures available (80.7% vs.73.2%), and there was no noted difference in blood pressure measure availability by age. Within the treated cohorts, both treatment cohorts had more than a year of follow-up on average (555 days for antimuscarinic patients, 456 days for mirabegron patients). A gap in coverage of at least 30 days was noted in 3.6% of antimuscarinic patients and 2.2% of mirabegron patients. Drop-out during the study period occurred in 34.1% of antimuscarinic patients (30.9% leaving plan, 3.2% death) and 21.1% of antimuscarinic patients (19.1% leaving plan, 2.0% death). Four percent of antimuscarinic patients switched treatment to mirabegron after index date, compared to 18.9% of mirabegron patients who had at least one antimuscarinic claim after index date. The result of the quality assessment process was a decision to proceed to the next phase of the study.

Baseline demographic characteristics prior to propensity matching, for both untreated and treated OAB patients, are shown in Table 1. Mirabegron patients tended to be older (mean age 70.1 years vs. 66.7, p<0.0001), were more likely to be male (33.6% vs. 26.8%, p = 0.01), Caucasian (88.7% vs. 85.9%, p = 0.24), and to be covered by supplementary Medicare (56.1% vs. 34.3%, p = 0.15), relative to antimuscarinic patients. The most notable difference between untreated and treated OAB patients was that the former group tended to be younger, with a mean age of 59.9 years, and 15.3% under age 40 years.

**Table 1:** Baseline demographic characteristics by OAB treatment group.

	Any OAB (N = 64,906)		Antimuscarinics (N = 10,311)		Mirabegron (N = 408)		Untreated (N = 54,187)		p-value (2 sided)***
Age at index date (years)									
Mean (SD)	61.1	17.0	66.7	13.7	70.1	12.5	59.9	17.3	<0.001*
Median (IQR)	64	50–75	69	58–78	73	62–80	62	48–74	
Category, n(%):									<0.001*
18–39	8,372	12.9	427	4.1	11	2.7	7,934	14.6	
40–59	18,722	28.8	2,459	23.8	61	15.0	16,202	29.9	
60–79	27,108	41.8	5,142	49.9	221	54.2	21,745	40.1	
≥80	10,704	16.5	2,283	22.1	115	28.2	8,306	15.3	
Sex, n (%)									0.010
Females	40,522	62.4	7544	73.16	271	66.42	32,731	50.4	
Males	24,357	37.5	2,764	26.8	137	33.6	21,456	39.6	
Sex unknown	27	0.0	3	0.0	0	0.0	24	0.0	
Race, n(%)									0.239
African American	4,903	7.6	800	7.8	22	5.4	4,081	7.5	
Asian	1,295	2.0	134	1.3	3	0.7	1,158	2.1	
Caucasian	53,380	82.2	8,862	85.9	362	88.7	44,156	81.5	
Other/ Unknown	5,328	8.2	515	5.0	21	5.1	4,792	8.8	
Ethnicity, n(%)									0.111
Hispanic	2,223	3.4	313	3.0	12	2.9	1,898	3.5	
Non-Hispanic	55,436	85.4	9,354	90.7	360	88.2	45,722	84.4	
Unknown	7,247	11.2	644	6.2	36	8.8	6,567	12.1	
Primary payer, n(%)									0.149
Commercial	36,471	56.2	5,568	54.0	146	35.8	13,327	24.6	
Medicaid	863	1.3	203	2.0	5	1.2	655	1.2	
Medicare	17,008	26.2	3,535	34.3	229	56.1	30,674	56.6	
Other Payor Type	1,236	1.9	176	1.7	4	1.0	1,056	1.9	
Uninsured	682	1.1	116						
Unknown	8,646	13.3	713	6.9	24	5.9	7,909	14.6	
Geographic area, n(%)**									<0.001*
East North Central	17,665	27.2	3,022	29.3	84	20.6	14,559	26.9	
East South Central	1,282	2.0	248	2.4	15	3.7	1,019	1.9	
Middle Atlantic	7,005	10.8	529	5.1	48	11.8	6,428	11.9	
Mountain	1,433	2.2	196	1.9	2	0.5	1,235	2.3	
New England	1,693	2.6	284	2.8	4	1.0	1,405	2.6	
Other/ Unknown	1,622	2.5	209	2.0	4	1.0	1,409	2.6	
Pacific	4,967	7.7	1,010	9.8	19	4.7	3,938	7.3	
South Atlantic/West South Central	19,357	29.8	3,481	33.8	196	48.0	15,680	28.9	
West North Central	9,882	15.2	1,332	12.9	36	8.8	8,514	15.7	
College education, n(%)*									<0.001*
< 14%	1,172	1.8	185	1.8	3	0.7	984	1.8	
14 to 18%	11,349	17.5	2,375	23.0	146	35.8	8,828	16.3	
19 to 28%	29,499	45.4	4,625	44.9	133	32.6	24,741	45.7	
>28%	21,257	32.8	2,915	28.3	122	29.9	18,220	33.6	
Education status unknown, n(%)	1,629	2.5	211	2.0	4	1.0	1,414	2.6	
Household income, mean (SD)*	$45,645	$11,838	$44,198	$10,776	$43,228	$11,435	$45,940	$12,012	0.093

N = number; SD = Standard deviation

* Mirabegron vs. antimuscarinics

**Recorded at the level of the 3-digit zip code

*** p-value calculated for the antimuscarinics and mirabegron treatment groups

Baseline unmatched clinical characteristics are reported in Table 2. Most distributions were similar across treatment groups. Differences among mirabegron patients include a higher rate of prior major adverse cardiovascular events (13.5% vs 11.7% in antimuscarinic patients and 10.1% in untreated patients in the year prior to baseline, p = 0.2687), and a higher rate of diabetes mellitus (42.4% vs 34.8% in antimuscarinic patients and 26.3% in untreated patients, p = 0.0021).

**Table 2:** Baseline clinical characteristics by OAB treatment group.

	Any OAB (N = 64,906)_		Antimuscarinics (N = 10,311)		Mirabegron (N = 408)		Untreated (N = 54,187)		p-value (2 sided)*
Current smoker, n(%)	6,051	9.3	1,158	11.2	40	9.8	4,853	9.0	0.209
Smoking status unknown	13,787	21.2	1,179	11.4	29	7.1	12,579	23.2	
BMI, mean (SD)	34.88	295.1	33.31	125.9	42.84	264.1	35.1	320.7	0.468
BMI, n(%)									
<18.5	828	1.3	141	1.4	6	1.5	681	1.3	0.001
18.5–24.9	14,998	23.1	2,308	22.4	90	22.1	12,600	23.3	
25.0–29.9	18,171	28.0	2,989	29.0	150	36.8	15,032	27.7	
≥30.0	20,943	32.3	4,367	42.4	155	38.0	16,421	30.3	
Unknown	9,966	15.4	506	4.9	7	1.7	9,453	17.4	
Most recent BP measure									
SBP, mean (SD)	126.5	17.6	128.4	17.9	128.9	17.4	126.0	17.5	0.570
DBP, mean (SD)	74.4	10.5	73.9	10.5	72.7	10.4	74.5	10.4	0.023
JNC-7 category, n(%)									
SBP<120 mmHg and DBP<80 mmHg	17,010	26.2	2,709	26.3	100	24.5	14,201	26.2	0.455
SBP 120–139 mmHg or DBP 80–89 mmHg	32,433	50.0	5,922	57.4	230	56.4	26,281	48.5	
SBP 140–159 mmHg or DBP 90–99 mmHg	6,572	10.1	1,374	13.3	65	15.9	5,133	9.5	
SBP ≥160 mmHg or DBP ≥100 mmHg	1,081	1.7	254	2.5	11	2.7	816	1.5	
Most recent cholesterol measure, mean (SD)									
HDL	53.8	16.8	53.8	16.73	53.60	17.1	53.86	16.88	0.817
LDL	104.7	348.4	100.6	33.86	97.12	34.5	105.71	389.28	0.046
HDL unknown, n(%)	28,746	44.3	3,326	8.2	153	1.5	25,267	46.6	0.939
LDL unknown, n(%)	28,781	44.3	3,317	8.1	155	1.5	25,309	46.7	
Any CV events prior to index date, n(%)									
MACE	6,747	10.4	1,208	11.7	55	13.5	5,484	10.1	0.269
non-MACE	4,979	7.7	842	8.2	38	9.3	4,099	7.6	0.427
Any (MACE or non-MACE)	8,209	12.6	1,478	14.3	70	17.2	6,661	12.3	0.102
Time since most recent event, mean (SD)									
MACE	637.2	631.2	580.2	576.4	647.1	684.2	649.6	641.6	0.0517
non-MACE	600.4	609.5	544.7	555.0	525.5	568.7	612.5	619.9	0.503
Any (MACE or non-MACE)	633.8	627.3	573.3	571.0	592.7	636.8	647.6	638.3	0.545
Number of recorded CV events prior to index date, mean(SD)									
MACE	0.6	2.7	0.6	3.0	0.7	3.0	0.54	2.7	0.509
non-MACE	0.4	2.3	0.4	2.4	0.4	2.3	0.38	2.3	1.000
Any (MACE or non-MACE)	0.8	3.5	0.9	3.8	1.0	3.8	0.77	3.5	0.602
Other CV comorbidities, n(%)									
Chronic kidney disease	6,972	10.7	1,593	15.4	59	14.5	5,320	9.8	0.621
Diabetes mellitus	17,992	27.7	3,589	34.8	172	42.2	14,231	26.3	0.002
Any prescription at index date, mean (SD)	1.8	1.5	2.2	1.9	1.6	1.5	1.5	1.0	p<0.001
History of OAB medication, n(%)									
Antimuscarinics	4,394	6.8	4,327	42.0	67	16.4	0	0.0	<0.001
Mirabegron	0	0.0	0	0.0	0	0.0	0	0.0	

BP = Blood pressure; CV = Cardiovascular; DBP = Diastolic blood pressure; HDL = High-density lipoprotein; LDL = Low-density lipoprotein; MACE = Major adverse cardiac event; N = number; SBP = Systolic blood pressure; SD = Standard deviation

* p-value calculated for the Antimuscarinics and mirabegron treatment groups

**Fig 2:**
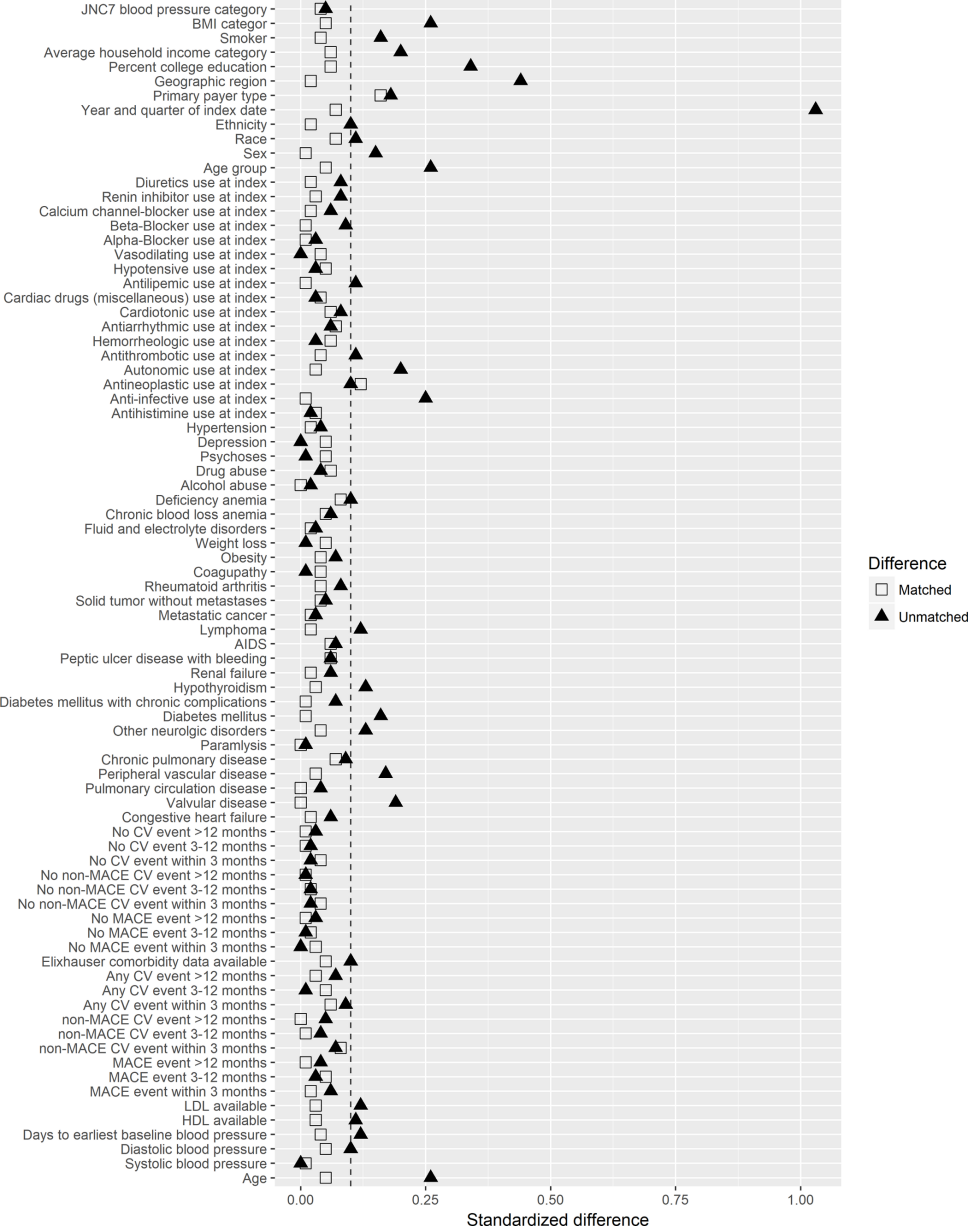
Standardized differences before and after propensity score matching.

The propensity score model included terms characterizing baseline demographics and clinical events, prior cardiovascular events, and pre-index blood pressure. After 3:1 propensity score matching, 396 of 408 mirabegron patients were matched to a corresponding sample of 1,188 antimuscarinic patients. The distributions of variables of interest before and after matching are reported in Table 3, and standardized differences before and after matching are shown in Fig 2. Prior to matching a number of variables were statistically different across the two treatment groups. Generally, mirabegron patients were more likely to have comorbidities, while antimuscarinic patients were more likely to be receiving concomitant medications. Propensity score matching was successful at reducing covariate imbalance across the treatment groups. After matching, no variables were statistically different between the two groups and all but one of the post-matching standardized difference was less than 0.10.

**Table 3:** Results of propensity score matching on demographic characteristics.

	Unmatched					3:1 propensity matched				
	Antimuscarinics (N = 10,311)		Mirabegron (N = 408)		p-value 2-sided	Antimuscarinics (N = 1,188)		Mirabegron (N = 396)		p-value 2-sided
	n	%	n	%		n	%	n	%	
Age group at index date										
18–39 yrs	427	4.1	11	2.7	<0.001	31	2.6	11	2.8	0.874
40–59 yrs	2459	23.9	61	15.0		188	15.8	61	15.4	
60–79 yrs	5142	49.9	221	54.2		666	56.1	215	54.3	
80 or older	2283	22.1	115	28.2		303	25.5	109	27.5	
Sex										
Female	7544	73.2	271	66.4	0.010	817	68.8	267	67.4	0.618
Male	2764	26.8	137	33.6		371	31.2	129	32.6	
Unknown	3	0.0	0	0.0						
Race										
African America	800	7.8	22	5.4	0.239	54	4.6	22	5.6	0.721
Asian	134	1.3	3	0.7		15	1.3	3	0.8	
Caucasian	8862	86.0	362	88.7		1056	88.9	351	88.6	
Other/Unknown	515	5.0	21	5.2		63	5.3	20	5.1	
Ethnicity										
Hispanic	313	3.0	12	2.9	0.111	41	3.5	12	3.0	0.919
Not Hispanic	9354	90.7	360	88.2		1047	88.1	351	88.6	
Unknown	644	6.3	36	8.8		100	8.4	33	8.3	
Primary payer										
Commercial	5568	54.0	229	56.1	0.149	650	54.7	218	55.1	0.356
Medicaid	203	2.0	5	1.2		23	1.9	5	1.3	
Medicare	3535	34.3	146	35.8		415	34.9	145	36.6	
Other Payor Type	176	1.7	4	1.0		10	0.8	4	1.0	
Uninsured	116	1.1	0	0.0		13	1.1	0	0.0	
Unknown	713	6.9	24	5.9		77	6.5	24	6.1	
Geographic area										
Midwest	4354	42.2	120	29.4	<0.001	359	30.2	120	30.3	0.999
Northeast	813	7.9	52	12.8		143	12.0	48	12.1	
Other/Unknown	209	2.0	4	1.0		14	1.2	4	1.0	
South	3729	36.2	211	51.7		608	51.2	203	51.3	
West	1206	11.7	21	5.2		64	5.4	21	5.3	
Percent college educated										
14 to 18%	2375	23.0	146	35.8	<0.001	443	37.3	139	35.1	0.903
19 to 28%	4625	44.9	133	32.6		378	31.8	132	33.3	
< 14%	185	1.8	3	0.7		6	0.5	3	0.8	
>28%	2915	28.3	122	29.9		347	29.2	118	29.8	
Unknown	211	2.1	4	1.0		14	1.2	4	1.0	
Average household income										
0K to 35K	2250	21.8	107	26.2	0.005	288	24.2	104	26.3	0.899
36K to 41K	3022	29.3	139	34.1		396	33.3	133	33.6	
42K to 51K	2241	21.7	79	19.4		237	20.0	78	19.7	
52K+	2587	25.1	79	19.4		253	21.3	77	19.4	
Unknown	211	2.1	4	1.0		14	1.2	4	1.0	
Year and quarter of index date										
01OCT2012	3685	35.7	14	3.4	<0.001	42	3.5	14	3.5	0.995
01JAN2013	1269	12.3	20	4.9		54	4.6	20	5.1	
01APR2013	798	7.7	41	10.1		133	11.2	40	10.1	
01JUL2013	753	7.3	50	12.3		140	11.8	49	12.4	
01OCT2013	685	6.6	58	14.2		169	14.2	53	13.4	
01JAN2014	1135	11.0	92	22.6		284	23.9	91	23.0	
01APR2014	750	7.3	59	14.5		154	13.0	57	14.4	
01JUL2014	622	6.0	32	7.8		96	8.1	32	8.1	
01OCT2014	614	6.0	42	10.3		116	9.8	40	10.1	
Falsification outcomes (post-index)										
Shingles	223	2.2	7	1.7	0.541	29	2.4	7	1.8	0.436
Hepatitis C	47	0.5	2	0.5	0.920	6	0.5	2	0.5	1.000
Hepatitis C or Shingles	269	2.6	9	2.2	0.616	35	3.0	9	2.3	0.480
CAP	1032	10.0	32	7.8	0.151	106	8.9	31	7.8	0.502
Body mass index category										
18.5–24.9	2310	22.4	91	22.3	<0.001	246	20.7	89	22.5	0.961
25.0–29.9	2994	29.0	151	37.0		442	37.2	143	36.1	
<18.5	136	1.3	6	1.5		17	1.4	6	1.5	
> = 30.0	4229	41.0	152	37.3		460	38.7	150	37.9	
Unknown	642	6.2	8	2.0		23	1.9	8	2.0	
Earliest baseline systolic blood pressure available										
12+ months	217	2.1	8	2.0	0.133	30	2.5	8	2.0	0.899
3 to <6 months	1507	14.6	47	11.5		144	12.1	45	11.4	
6 to <9 months	682	6.6	22	5.4		68	5.7	21	5.3	
9 to <12 months	414	4.0	11	2.7		40	3.4	11	2.8	
<3 months	7491	72.7	320	78.4		906	76.3	311	78.5	
Earliest baseline diastolic blood pressure available										
12+ months	217	2.1	8	2.0	0.132	30	2.5	8	2.0	0.899
3 to <6 months	1507	14.6	47	11.5		144	12.1	45	11.4	
6 to <9 months	683	6.6	22	5.4		68	5.7	21	5.3	
9 to <12 months	414	4.0	11	2.7		40	3.4	11	2.8	
<3 months	7490	72.6	320	78.4		906	76.3	311	78.5	
JNC7										
Other	60	0.6	3	0.7	0.963	10	0.8	3	0.8	0.986
SBP 120–139 or DBP 80–89 mmHg	5910	57.3	239	58.6		679	57.2	230	58.1	
SBP 140–159 or DBP 90–99 mmHg	1374	13.3	56	13.7		173	14.6	56	14.1	
SBP >160 or DBP >100 mmHg	220	2.1	7	1.7		26	2.2	7	1.8	
SBP<120 and DBP<80 mmHg	2745	26.6	103	25.3		300	25.3	100	25.3	
Unknown	2	0.0	0	0.0						
Baseline cholesterol available										
HDL	6995	67.8	255	62.5	0.024	736	62.0	251	63.4	
LDL	7033	68.2	255	62.5	0.015	736	62.0	251	63.4	0.611
Cardiovascular events prior to index date										
MACE event <3 months prior	232	2.3	13	3.2	0.215	35	3.0	13	3.3	0.735
MACE event 3–12 months prior	403	3.9	18	4.4	0.608	43	3.6	18	4.6	0.407
MACE event >12 months prior	816	7.9	37	9.1	0.398	111	9.3	36	9.1	0.881
Non MACE event <3 months prior	149	1.5	10	2.5	0.099	17	1.4	10	2.5	0.145
Non MACE event 3–12 months prior	287	2.8	9	2.2	0.485	28	2.4	9	2.3	0.924
Non MACE event >12 months prior	587	5.7	28	6.9	0.319	77	6.5	26	6.6	0.953
Any MACE event <3 months prior	307	3.0	19	4.7	0.053	43	3.6	19	4.8	0.295
Any MACE event 3–12 months prior	531	5.2	22	5.4	0.828	53	4.5	22	5.6	0.375
Any MACE event >12 months prior	1004	9.7	48	11.8	0.177	128	10.8	46	11.6	0.643
Comorbidities										
Congestive heart failure	1096	10.6	51	12.5	0.231	144	12.1	46	11.6	0.789
Valvular disease	1641	15.9	95	23.3	<0.001	262	22.1	87	22.0	0.972
Pulmonary circulation disease	561	5.4	26	6.4	0.417	70	5.9	23	5.8	0.951
Peripheral vascular disease	1876	18.2	102	25.0	<0.001	257	21.6	91	23.0	0.575
Hypertension	6571	63.7	268	65.7	0.420	754	63.5	256	64.7	0.673
Paralysis	508	4.9	19	4.7	0.805	56	4.7	19	4.8	0.946
Other neurological disorders	2173	21.1	109	26.7	0.006	279	23.5	100	25.3	0.475
Chronic pulmonary disease	2736	26.5	124	30.4	0.084	325	27.4	120	30.3	0.259
Diabetes w/o chronic complications	2856	27.7	144	35.3	0.001	401	33.8	135	34.1	0.902
Diabetes w/ chronic complications	1271	12.3	60	14.7	0.153	167	14.1	57	14.4	0.868
Hypothyroidism	2346	22.8	115	28.2	0.011	311	26.2	109	27.5	0.599
Renal failure	1275	12.4	43	10.5	0.271	123	10.4	43	10.9	0.776
Liver disease	517	5.0	21	5.2	0.904	61	5.1	21	5.3	0.896
Peptic ulcer Disease x bleeding	32	0.3	3	0.7	0.140	2	0.2	2	0.5	0.248
Acquired immune deficiency syndrome	25	0.2	0	0.0	0.319	2	0.2	0	0.0	0.414
Lymphoma	123	1.2	12	2.9	0.002	30	2.5	11	2.8	0.784
Metastatic cancer	280	2.7	9	2.2	0.533	24	2.0	9	2.3	0.761
Solid tumor w/out metastasis	1710	16.6	76	18.6	0.277	203	17.1	73	18.4	0.541
Rheumatoid arthritis/collagen vas	1037	10.1	52	12.8	0.078	130	10.9	48	12.1	0.520
Coagulopathy	517	5.0	21	5.2	0.904	67	5.6	19	4.8	0.522
Obesity	1880	18.2	64	15.7	0.190	207	17.4	63	15.9	0.487
Weight loss	869	8.4	33	8.1	0.809	83	7.0	33	8.3	0.373
Fluid and electrolyte disorders	2047	19.9	86	21.1	0.543	226	19.0	79	20.0	0.686
Chronic blood loss anemia	279	2.7	15	3.7	0.239	29	2.4	13	3.3	0.367
Deficiency anemia	2651	25.7	123	30.2	0.045	306	25.8	116	29.3	0.168
Alcohol abuse	199	1.9	7	1.7	0.757	18	1.5	6	1.5	1
Drug abuse	236	2.3	12	2.9	0.390	25	2.1	12	3.0	0.291
Psychoses	1597	15.5	65	15.9	0.808	169	14.2	64	16.2	0.346
Depression	2543	24.7	101	24.8	0.966	274	23.1	99	25.0	0.432
Index medications										
History of prior antimuscarinic use	4327	42.0	67	16.4	<0.001	229	19.3	67	16.9	0.297
Antihistamine Drugs	39	0.4	2	0.5	0.719	4	0.3	2	0.5	0.637
Anti-infective Agents	851	8.3	11	2.7	<0.001	34	2.9	11	2.8	0.930
Antineoplastic Agents	52	0.5	0	0.0	0.151	8	0.7	0	0.0	0.102
Autonomic Drugs	629	6.1	9	2.2	0.001	22	1.9	9	2.3	0.601
Antithrombotic Agents	242	2.4	4	1.0	0.071	17	1.4	4	1.0	0.526
Hematopoietic Agents	1	0.0	0	0.0	0.842					
Hemorrheologic Agents	5	0.1	0	0.0	0.656	2	0.2	0	0.0	0.414
Antihemorrhagic Agents	1	0.0	0	0.0	0.842					
Antiarrhythmic Agents	19	0.2	0	0.0	0.386	3	0.3	0	0.0	0.317
Cardiotonic Agents	33	0.3	0	0	0.252	2	0.2	0	0.0	0.414
Cardiac Drugs, Miscellaneous	6	0.1	0	0	0.626	1	0.1	0	0.0	0.564
Antilipemic Agents	926	9.0	25	6.13	0.047	75	6.3	24	6.1	0.857
Hypotensive Agents	45	0.4	1	0.25	0.562	7	0.6	1	0.3	0.413
Vasodilating Agents	80	0.8	3	0.74	0.927	13	1.1	3	0.8	0.562
Alpha-Adrenergic Blocking Agents	48	0.5	3	0.74	0.437	7	0.6	2	0.5	0.847
Beta-Adrenergic Blocking Agents	517	5.0	13	3.19	0.095	41	3.5	13	3.3	0.873
Calcium-Channel Blocking Agents	416	4.0	12	2.94	0.269	33	2.8	12	3.0	0.793
Renin-Angiotensin-Aldosterone System Inhibitors	813	7.9	24	5.88	0.139	62	5.2	23	5.8	0.652
Diuretics	434	4.2	11	2.7	0.133	38	3.2	11	2.8	0.675

CAP = Community-acquired pneumonia; DBP = Diastolic blood pressure; HDL = High-density lipoprotein; JNC-7 = Seventh report of the joint national committee on prevention, detection, evaluation, and treatment of high blood pressure; LDL = Low-density lipoprotein; MACE = Major adverse cardiac event; SBP = Systolic blood pressure

**Fig 3:**
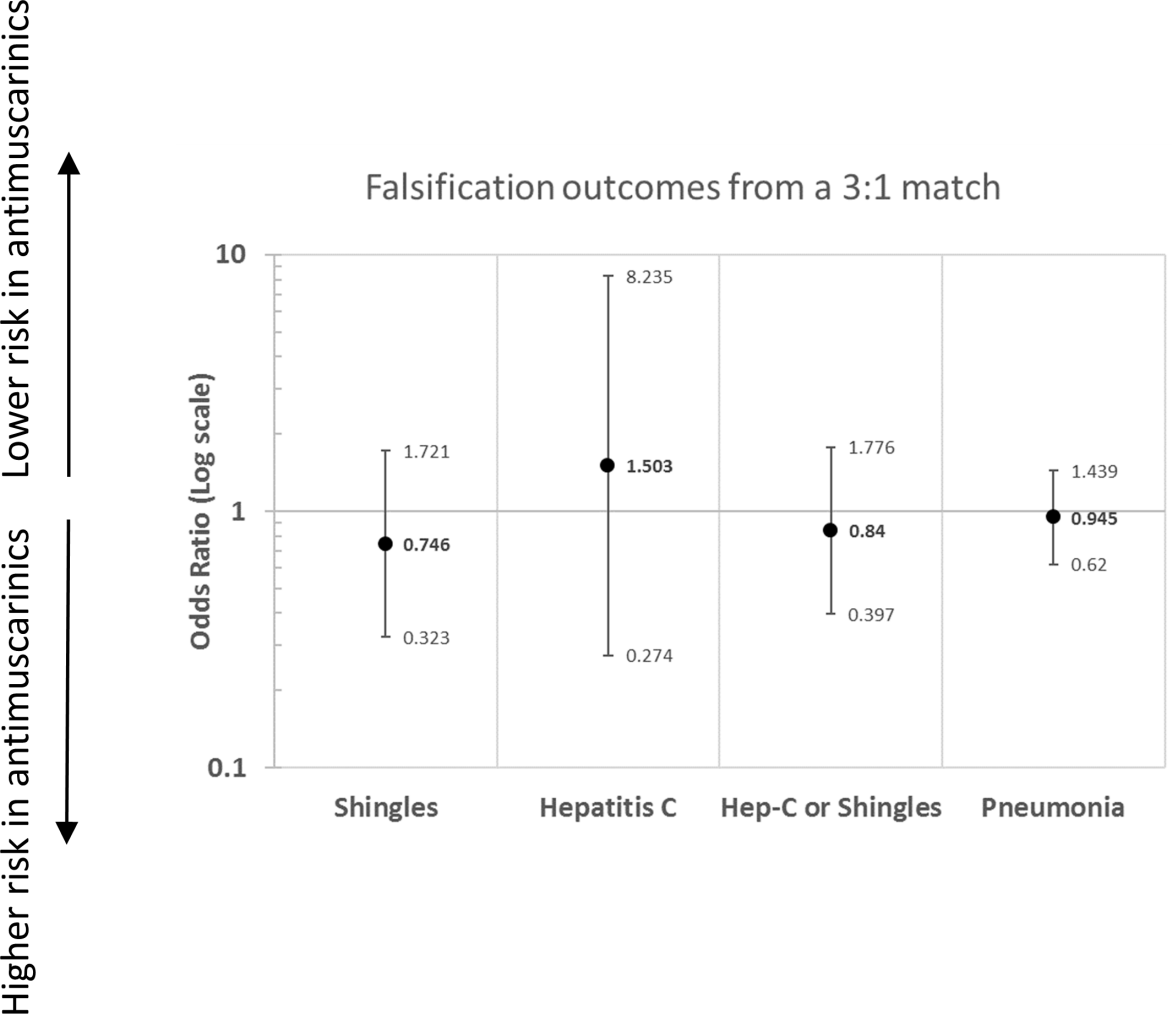
Results of falsification analysis.

The results of the falsification analysis on the propensity-matched population are shown in Fig 3. Due to the relative infrequency of shingles and hepatitis C virus, they were combined *post hoc* into a composite endpoint. There was no statistical evidence of a spurious association between any of the falsification endpoints and mirabegron or antimuscarinic treatment (all 95% confidence intervals span 1.0). However, the confidence intervals were wide, particularly for hepatitis C (point estimate 1.5, 95% confidence interval 0.27–8.2). Thus, due in part to the low sample size contributing to a lack of power, the presence of residual bias (as evidenced by an odds ratio different than 1.0) in the propensity-matched dataset could not be ruled out.

## Discussion

This study aimed to characterize the cardiovascular risk profile of untreated, mirabegron-treated, and antimuscarinic-treated OAB patients, using an integrated claims/EHR dataset. An incremental analytical approach was implemented to ensure rigor and accuracy within the constraints of observational data. In a retrospective cohort of individuals with OAB, mirabegron patients were found to be older, with more comorbidities and more prior cardiovascular events relative to antimuscarinic patients. Mirabegron is typically prescribed as a second-line agent to antimuscarinics, due to either inadequate response or poor tolerability of antimuscarinics, or due to formulary rules e.g. stepped therapy conditions. As such, unadjusted comparisons of treatment effectiveness or safety between mirabegron and antimuscarinics based on these data may be biased by differences in the distribution of baseline risk factors between treatment cohorts. Indeed, despite what appeared to be adequate propensity score matching, when residual bias was assessed through falsification analyses, the resulting confidence intervals were sufficiently wide that associations with falsification outcomes (as evidenced by wide intervals) could not be ruled out. The small sample size ultimately eligible for study inclusion–due to the significant attrition caused by lack of overlap of subjects between the claims and EHR datasets, and application of the relatively limited exclusion criteria–contributed to limited power and to confidence interval width, preventing a definitive interpretation of results and conclusions.

Bias assessment for the cardiovascular outcomes study, the initiation of which would be based on the results of the feasibility assessment, was defined with two pre-specified stopping rules with respect to data quality and study feasibility. The first stopping rule instructed no further analyses if there was evidence of sufficiently implausible data (i.e. clinical data values that were physically impossible and assumed to be data entry errors, assessed on a case-by-case basis) or frequency of missing data as assessed by the initial data quality assessment. The second stopping rule was a determination of feasibility after propensity-score matching, based on achievement of covariate balance, available sample size, and results of falsification analysis. No assessment of primary or secondary endpoints for the cardiovascular outcomes study was conducted prior to confirming the results of the stopping rules, to ensure that outcome values had no influence on the decision to continue the study. In accordance with the second analytic stopping rule, this, together with the potential for residual bias identified by the falsification analyses, led to the conclusion that there was insufficient evidence to rule out bias in the available data, and that the cardiovascular outcomes study could not be robustly carried out at this time. Future real-world administrative database studies assessing clinical outcomes across OAB treatments will require careful accounting for the clinical differences inherent in comparing populations receiving a second-line agent to a first-line agent beyond that achieved by standard methods such as propensity scoring; data sources with larger sample sizes available may mitigate the limitations caused by low power observed here. By planning the cardiovascular outcomes study with pre-specified stopping rules, primary and secondary endpoint results data were not analyzed in any way prior to making stopping decisions for the study. This ensured information about results could not influence the decision of whether to proceed with further analyses.

Electronic health records have been used previously to assess blood pressure outcomes,[14, 25–29] and are acknowledged by the US Food and Drug Administration for use in prospective clinical investigations of medications.[30] Integrated claims and EHR datasets can also be valuable research tools to assess clinical and pharmacoepidemiologic questions due to large sample sizes, long follow-up durations, and the inclusion of patients with complex medical needs who would not be likely candidates for clinical trial participation and cannot feasibly be assessed within an RCT framework. Indeed, in the search for robust real-world data, claims and EHR data sources are often cited as a powerful source of evidence, and the availability of large sample sizes is a frequently-noted feature.[31] That said, as reported here, rigorously identifying the study cohort and appropriately controlling for potential biases may impact the feasibility of using these data for complex clinical and pharmacoepidemiologic questions. Indeed, this study highlights that even for a relatively common condition such as OAB (with a study requirement for recorded claims data), sample size remained a limiting factor within a large administrative dataset.

With respect to the potential for selection bias in observational data sources, supplementing propensity scores with statistical techniques such as falsification analysis (also referred to as negative control analysis),[32] can help to assess whether an unbiased comparison is feasible with the available data, and can provide confidence in the interpretation of results. However, identification of appropriate endpoints can be challenging; in the case of OAB, the potential for an association between exposure and cardiovascular risk factors limits the availability of possible falsification endpoints with no plausible chance of association to treatment group. As a result, although expected frequency was a criterion used when selecting candidate falsification endpoints, the sample size was inadequate to conduct a conclusive falsification analysis. This highlights the importance of a large sample size, not only for attaining sufficient statistical power required for primary analyses, but also for bias assessment techniques. The methods described here may be used as a framework for other investigators who are considering real-world data to investigate other clinical and pharmacoepidemiologic research questions.

While data quality checks did not identify any notable concerns regarding data quality or conclusive evidence of residual bias, the magnitude of variability in falsification analysis results could not conclusively rule out the potential for residual bias in the sample after statistical adjustment. The most important concern with the data, eventually leading to the decision to not proceed with analysis, was the small sample of eligible mirabegron patients, including the attrition induced by individuals who were prescribed therapy but did not fill their prescription. In particular, the low number of patients relative to available mirabegron clinical trial populations, for which a similar length of follow-up is available for samples of 400–800 patients,[33–36] did not justify the potential for additional biases associated with observational research. It was notable that, of patients identified as receiving mirabegron or an antimuscarinic in EHR data, more than half were excluded for not having corresponding claims records. This may reflect problems with primary adherence to OAB medications, with patients choosing not to access prescribed medications; however, this feature of the data also highlights a potential limitation with linked EHR and claims data generally, in that dispensed prescriptions may not be reflected in claims data due to plan discontinuation and/or other changes in coverage.

While the relatively small sample size of mirabegron patients coupled with the low prevalence of the falsification endpoints of interest did not directly lead to the decision to stop the cardiovascular outcomes study, it may have contributed to the interpretation that falsification endpoints were unable to definitively rule out residual bias, as determined by the wide confidence intervals rather than point estimates. As such, a similar analysis in a larger sample may have been found sufficient to warrant continuation with the cardiovascular outcomes study. A recently-conducted US Food and Drug Administration mini-sentinel study acknowledged similar limitations; they did not find a difference in risk of acute myocardial infarction or stroke between new users of mirabegron vs. oxybutynin, while noting limitations due to available follow-up time and sparseness of outcomes.[37]

While long-term follow up of the real-world safety and effectiveness profiles of mirabegron and antimuscarinics warrants further consideration, the limited scope of available data is challenging for comparative analyses. The methodology presented here describes a framework for any treatment comparative analysis using real world observational data where treatment attributes may be at risk of residual confounding bias.

## Appendix A: Sample size calculation

Using a repeated measures analysis of variance (RM-ANOVA) carried out over 4 time points (0, 3, 6, and 12 months), sample sizes needed to detect clinically relevant differences in SBP/DBP were calculated on the basis of a global hypothesis that tests for equality between the two treatment groups at every time point.[36] This global test is appropriate whenever there are a wide variety of hypotheses of interest, including, for example, treatment differences at one or more time points, or comparisons between the change scores from one set of time points to another, etc. In this test, protection against a Type I error is maintained at the prescribed alpha-level for all such comparisons.[38] To err on the conservative side, the Type I error for comparing mean change from baseline at 3, 6 and 12 months for the co-primary endpoints of change in systolic and diastolic blood pressures was initially set at 0.05/3 = 0.0167 so as to yield an overall Bonferroni-adjusted Type I error of 0.05. Power was set at 0.90. Under conservative assumptions regarding correlation[38] and assuming 1:1 matching of patients, sample size calculations showed that detecting a systolic blood pressure difference of 2.5 mmHg requires 1,117 individuals in each treatment group assuming a 14 mm Hg standard deviation, and 2,277 individuals in each treatment group assuming a 20 mm Hg standard deviation.[39] However, based on a lower than anticipated number of eligible mirabegron patients relative to antimuscarinic patients, revised sample size calculations were made based on a 3:1 matching of antimuscarinic to mirabegron patients with the Type I error set at 0.05. At power of 0.80, a sample of 500 mirabegron patients and 1500 antimuscarinic patients would be required to detect a systolic blood pressure difference of 2.5 mmHg assuming a 14 mmHg standard deviation. When based on a power of 0.90, the sample size increased to 645 mirabegron patients and 1935 antimuscarinic patients.
